# A Preliminary Report Regarding the Morphological Changes of Nano-Enabled Pharmaceutical Formulation on Human Lung Carcinoma Monolayer and 3D Bronchial Microtissue

**DOI:** 10.3390/medicina60020208

**Published:** 2024-01-25

**Authors:** Cătălin Prodan-Bărbulescu, Claudia-Geanina Watz, Elena-Alina Moacă, Alexandra-Corina Faur, Cristina-Adriana Dehelean, Flaviu Ionut Faur, Laura Octavia Grigoriţă, Anca Laura Maghiari, Paul Tuţac, Ciprian Duţă, Sorin Bolintineanu, Laura Andreea Ghenciu

**Affiliations:** 1 Department I—Discipline of Anatomy and Embryology, Faculty of Medicine, “Victor Babeş” University of Medicine and Pharmacy Timisoara, 2nd Eftimie Murgu Square, RO-300041 Timisoara, Romania; catalin.prodan-barbulescu@umft.ro (C.P.-B.); faur.alexandra@umft.ro (A.-C.F.); grigorita.laura@umft.ro (L.O.G.); boscuanca@yahoo.com (A.L.M.); s.bolintineanu@umft.ro (S.B.); bolintineanu.laura@umft.ro (L.A.G.); 2 Faculty of Pharmacy, “Victor Babeş” University of Medicine and Pharmacy Timisoara, 2nd Eftimie Murgu Square, RO-300041 Timisoara, Romania; farcas.claudia@umft.ro (C.-G.W.); cadehelean@umft.ro (C.-A.D.); 3 Research Centre for Pharmaco-Toxicological Evaluation, “Victor Babes” University of Medicine and Pharmacy Timisoara, 2nd Eftimie Murgu Square, RO-300041 Timisoara, Romania; 4 Department X—Discipline of Surgery II, Faculty of Medicine, “Victor Babeş” University of Medicine and Pharmacy Timisoara, 2nd Eftimie Murgu Square, RO-300041 Timisoara, Romania; flaviu.faur@umft.ro (F.I.F.); duta.ciprian@umft.ro (C.D.); 5 2nd Surgery Clinic, “Pius Brinzeu” Clinical Emergency County Hospital, RO-300723 Timisoara, Romania; 6 Toxicology and Molecular Biology Department, “Pius Brinzeu” Clinical Emergency County Hospital, RO-300723 Timisoara, Romania; paul.tutac@gmail.com; 7 Department III—Discipline of Physiopathology, Faculty of Medicine, “Victor Babeş” University of Medicine and Pharmacy Timisoara, 2nd Eftimie Murgu Square, RO-300041 Timisoara, Romania

**Keywords:** *Camellia sinensis*, *Ocimum basilicum*, magnetite, maghemite, A549, EpiAirway^TM^

## Abstract

*Background and Objectives*: Nowadays, the development of enabled pharmaceutical nanoparticles of solid lipid type is continuously growing, because they have the potential to be used for targeted drug release leading to an increased effect of chemotherapy, being used in lung cancer nano-diagnosis and nano-therapy. The current study reports the preliminary results obtained regarding the biological effect of a new nano-enabled pharmaceutical formulation in terms of its cytotoxic and biosafety profile. *Materials and Methods*: The pharmaceutical formulations consist of solid lipid nanoparticles (SLN) obtained via the emulsification–diffusion method by loading green iron oxide nanoparticles (green-IONPs) with a pentacyclic triterpene (oleanolic acid—OA). Further, a complex biological assessment was performed, employing three-dimensional (3D) bronchial microtissues (EpiAirway^TM^) to determine the biosafety profile of the SLN samples. The cytotoxic potential of the samples was evaluated on human lung carcinoma, using an in vitro model (A549 human lung carcinoma monolayer). *Results*: The data revealed that the A549 cell line was strongly affected after treatment with SLN samples, especially those that contained OA-loaded green-IONPs obtained with *Ocimum basilicum* extract (under 30% viability rates). The biosafety profile investigation of the 3D normal in vitro bronchial model showed that all the SLN samples negatively affected the viability of the bronchial microtissues (below 50%). As regards the morphological changes, all the samples induce major changes such as loss of the surface epithelium integrity, loss of epithelial junctions, loss of cilia, hyperkeratosis, and cell death caused by apoptosis. *Conclusions*: In summary, the culprit for the negative impact on viability and morphology of 3D normal bronchial microtissues could be the too-high dose (500 µg/mL) of the SLN sample used. Nevertheless, further adjustments in the SLN synthesis process and another complex in vitro evaluation will be considered for future research.

## 1. Introduction

Nowadays, nanotechnology plays a vital role worldwide due to the benefits and potential it can provide to humans. A few decades ago, nanotechnology was implemented in industry, but this science has progressively influenced various areas of material applications, including the medical area [1]. In the biomedical field, nanotechnology offers many benefits regarding the treatment of various human diseases, taking into account precise target-oriented drug delivery to a specific organ. Generally, nanotechnology involves the study of well-defined nanostructures, by the association of nanoparticles under 100 nm with basic building blocks of different dimensions at the molecular level. Nanoparticles exhibit the great advantage of consisting in a small size and high surface area-to-volume ratio with the property of conjugation/encapsulation of different coating materials, such as organic polymers, organic surfactants, inorganic metals, inorganic compounds, or bioactive molecules [2]. Of all known structured nano-carriers, lipid nanostructures offer the possibility for developing novel therapeutics formulations, due to their unique and tailorable-dependent properties. Lipids were promoted as alternative carriers, especially for lipophilic pharmaceutical products. Lipid nanoparticles known as solid lipid nanoparticles (SLNs) have attracted the attention of researchers because they improve the stability of pharmaceutical products and are also able to carry both lipophilic and hydrophilic drugs [3,4]. Compared to other colloidal carriers (polymeric, emulsions, liposomes), SLNs show many advantages, such as excellent biocompatibility (no toxicity because of the physiological biodegradable lipids), biodegradability, a less expensive fabrication process, sterilizability, easily industrially scaleable production, long-term stability, high entrapment efficiency, the association with the majority of molecules, and so on [5,6]. In addition to the aforementioned advantages, if they carry lipophilic drugs, the latter are automatically protected from the degradation process, because they are entrapped into the SLN lipid nanostructure [7]. However, SLNs possess also several disadvantages, namely poor drug loading capacity, a polymeric transition during storage which may lead to drug expulsion, rapid clearance, unexpected crystallization of drugs, the high water content of the dispersions (leads to formulation instability), and nonspecific uptake by the mononuclear phagocytic system [8,9,10]. Nevertheless, by using SLNs as nanocarriers, drug administration via different routes is increased (transdermal, parenteral, oral, nasal, ocular, pulmonary, rectal, or vaginal) [3,4,11]. Due to their nanometric scale and other unique properties they present [12,13,14,15,16,17], the SLN can form a large surface that facilitates the attachment of various ligands (antibodies, aptamers, peptides, DNA, RNA) [18,19,20,21,22]. Therefore, SLNs are suitable candidates mainly applied in targeted drug delivery approaches.

Lung cancer represents the second most common type of cancer worldwide, but it is the first type in the leading causes of cancer-related deaths [23]. According to the GLOBOCAN 2023 database (https://gco.iarc.fr/today/home accessed on 5 November 2023), published by the International Agency for Research on Cancer (IARC), in 2020 in Romania, the number of new cases of lung cancer was over 12,000 new cases, comprising both sexes and all ages, ranked second place after colorectal cancer, with twofold higher incidence in males than in females [24]. The determinant factors that contribute to lung cancer development include hookah smoking or cigarettes, exposure to toxins, and air pollution [25,26,27]. Conventional treatment alternatives for lung cancer include surgery, chemotherapy, and radiotherapy. For the advanced metastatic stages of the disease, targeted therapy or/and immunotherapy are the treatment options included [28]. Although new advances in the treatment of lung cancer have been developed [23], new treatments for patients suffering from end-stage lung cancer and beyond are still needed. Several strategies regarding enhanced drug release in tumor cell cytoplasm as well as nanotechnology-enabled formulations have been investigated because combined chemotherapy is not able to enrich only the tumor area [29]. Nanotherapeutics can induce cancer-specific cytotoxicity and modulate antitumor immunity by targeting the tumor, thus enhancing the treatment and reducing adverse reactions [30]. Therefore, the development of enabled pharmaceutical nanoparticles has the potential to be used for targeted drug release, leading to an increased effect of chemotherapy in lung cancer [31]. Solid lipid nanoparticles are the desired nano-enabled formulation to deliver anticancer drugs to the tumor site, being used in lung cancer nano-diagnosis and nano-therapy [32].

The most desired nanoparticles used as specific-targeted drug delivery vectors are magnetic iron oxide nanoparticles (IONPs). Due to their unique physicochemical features (strong magnetic moment and superparamagnetic behavior, bioavailability and biocompatibility with living organisms, low toxicity, large surface area suitable for conjugating or encapsulating other compounds, and nanoscale size), these nanosystems are considered an alternative cancer treatment approach for targeted non-small cell lung cancer (NSCLC) therapy [33,34,35,36,37,38,39,40,41,42,43,44,45,46,47,48]. The studies mentioned above demonstrate promising results regarding the use of IONPs in cancer diagnoses or as drug delivery systems, yet no study has demonstrated an efficient therapy in humans. One of the great disadvantages of the above-mentioned nanoparticles is the synthesis method, which plays a crucial role in obtaining magnetic IONPs with the desired features. Green synthesis is an eco-friendly, simple, and inexpensive method, based on the use of bacteria, fungi, yeast, algae, viruses, actinomycetes, or plant extracts, with high medicinal importance in obtaining green-IONPs with well-defined composition, surface chemistry, size, and shape. The biological entities used will act in the synthesis process as both reducing and stabilizing agents [49].

Many studies report the incorporation of different drugs into the SLN structure, demonstrating their abilities as anticancer drug delivery nano-platforms, used instead of anticancer drugs that are normally refractory to cytotoxic drug treatment [8]. However, as far as we know, no study reports the use of green IONPs as a nanocarrier for oleanolic acid as a solid lipid pharmaceutical formulation investigated for its potential application in lung cancer. Based on these aspects, the main goal of the present research was to develop a new multifunctional nanostructure with both antitumor-inducing and remote drug delivery characteristics able to release the loaded antitumor drug under normal conditions. Oleanolic acid (OA) is a pentacyclic triterpene known to possess a wide range of pharmacological effects, including anti-inflammatory, antiviral, hepatoprotective, antiangiogenic, and antitumoral effects [50]. It is expected that the obtained SLN formulation manifests a synergic effect, due to the combination of OA biological effects with plant phytocompounds, because the main polyphenols originate from the plant extract used in the synthesis of green IONPs and remain at nanoparticle surfaces (as stabilizing agents), being in direct contact with the anticancer drug (OA) [51,52]. Therefore, the main objective for the development of a nano-enabled pharmaceutical formulation was the addressability of green-IONPs for biomedical applications, especially as drug delivery nanocarriers.

Following the above, the current study reports a facile, single-step, and cost-effective method for the synthesis of a novel nano-enabled pharmaceutical formulation based on OA-loaded green IONPs, developed starting from two ethanolic extracts of *Camellia sinensis* and *Ocimum basilicum* plant materials. Obtaining a solid lipid nanoparticle (SLN) nanoformulation allows us to carry out a preliminary study regarding its in vitro biological assessment on human lung carcinoma. In this regard, four different SLNs were obtained, which all were further analyzed in terms of shape, size, hydrodynamic diameter, polydispersity index, and zeta potential, by employing physicochemical analyses. In addition, a complex biological assessment was performed, by employing three-dimensional (3D) bronchial microtissues (EpiAirway^TM^ model, MatTek Corporation, Bratislava, Slovak Republic) to determine the biosafety profile of the SLN test samples, while the evaluation of the cytotoxic potential of the samples on human lung carcinoma was realized by using an in vitro model based on an A549 human lung carcinoma monolayer.

## 2. Materials and Methods

### 2.1. Preparation of SLN Test Samples

The solid lipid nanoparticle (SLN) test samples consist of iron oxide nanoparticles (IONPs) obtained by green synthesis, followed by loading them with a pentacyclic triterpene–oleanolic acid (OA). The fabrication of green iron oxide nanoparticles (green-IONPs) was started from 2 alcoholic extracts based on *Camellia sinensis* and *Ocimum basilicum* plant materials. It was established that the fabrication process occurs at two different temperatures: at 25 °C and 80 °C, respectively. Thus, four green-IONP samples were obtained, which were noted as CS 25, CS 80, OB 25, and OB 80. The detailed protocol regarding the preparation of the extracts as well as the protocol regarding the fabrication of green-IONPs are extensively explained in previously reported research effectuated by Moaca and co-workers [53].

After the green-IONPs were obtained and physicochemically characterized (results reported also in reference [53]), these were used for SLN fabrication. The OA-loaded green IONPs were fabricated by the emulsification–diffusion method according to the preparation technique described by Oliveira and co-workers [54]. The first time, we obtained an organic phase containing 25.6 mg phosphatidylcholine dissolved in ethyl alcohol (9 mL). This organic phase was added to another organic phase consisting of 20 mg green-IONPs, 40 mg glyceryl monostearate, and 1 mg of OA dissolved in 5 mL of acetone. Subsequently, these two organic phases were added to an aqueous phase containing 4 mg Span 80 dissolved in 25 mL ultrapure water provided by a MiliQ system (Merck Millipore, Darmstadt, Germany). The organic solvent was eliminated by evaporation using a rotary vacuum evaporator under reduced pressure (Laborata 4000eco from Heidolph Instruments GmbH & Co. KG, Schwabach, Germany). Then, the samples were subjected to the sonication process (50% amplitude) in an ice bath, using a Q700 Sonica ultrasound device (Newtown, CT, USA). After 15 min of sonication, the SLNs based on OA-loaded green IONPs were formed and noted for further identification as SLN_CS 25@OA, SLN_CS 80@OA, SLN_OB 25@OA, and SLN_OB 80@OA. The samples were cooled down at room temperature (22 ± 2 °C) and refrigerated until further use.

**Figure 1:**
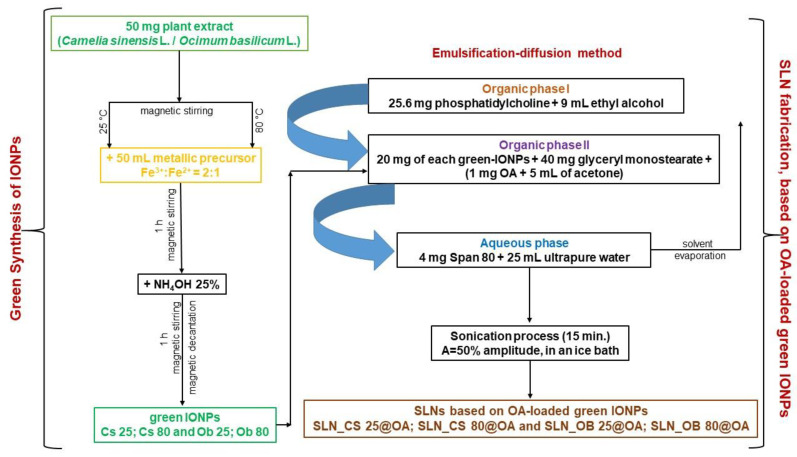
Schematic protocol of the experimental design.

For a better understanding of the entire process of sample preparation, Figure 1 depicts the schematic protocol of the experimental design.

### 2.2. SLN Characterization

#### 2.2.1. Determination of the Entrapment Efficiency (EE%) and Drug-Loaded Capacity (DLC%) of the OA into the SLNs Based on OA-Loaded Green IONPs

The amount of oleanolic acid (OA) was determined by a reversed-phase HPLC method using a 6120 LC-MS analytical system from Agilent (Santa Clara, CA, USA). The system was equipped with an electrospray ionization source (ESI) and consisted of a 1260 Infinity HPLC coupled with a Quadrupolar (Q) mass spectrometer. The column used was a Zorbax Eclipse Plus C18 column (3.0 mm × 100 mm × 3.5 μm), and the column temperature was kept constant at 40° ± 0.5 °C. A mixture of 85% methanol (obtained from Merck, Darmstadt, Germany) and 15% ammonium formate of 1 mM (from Agilent, Santa Clara, CA, USA) in isocratic elution was used as the mobile phase. UV detection was carried out at 210 nm, with a flow rate of 1.0 mL/min at 25 °C and an injection volume of 20 μL [55]. An external calibration curve was obtained using a 7-point plot in the 10–2500 ng/mL range (R^2^ > 0.9996 linearity) and was used for the sample quantification.

The entrapment efficiency (EE%) of OA was calculated by estimating the concentration of the free drug in the liquid phase; thus, the SLNs based on OA-loaded green IONPs were centrifuged at 10,000 rpm for 10 min using a ThermoMicro CL17 microcentrifuge (Thermo Fisher Scientific, Waltham, MA, USA). The supernatant (1000 μL) was collected and dissolved in 5 mL of mobile phase and analyzed using HPLC with UV detection. The EE% was calculated using the following formula:

(1) $$EE\%=\frac{OA_{total}-OA_{free}}{OA_{total}}\times 100\%$$

where $OA_{total}$ is the total weight of the OA, and $OA_{free}$ is the free OA content present in the liquid phase.

The quantitative determination of the OA-loading capacity (DLC%) of the SLNs based on OA-loaded green IONPs was performed using the lipid precipitation method. Briefly, 500 μL of SLN-based OA-loaded green IONPs was dissolved in 5 mL methanol and filtrated through a 0.22 μm Whatman membrane filter (Sigma–Aldrich, St. Louis, MO, USA). Then, the mixture was centrifuged at 10,000 rpm for 10 min followed by the dilution of 1000 μL supernatant in 5 mL of mobile phase. The solution was analyzed using the validated HPLC method with UV detection at 210 nm, according to the chromatographic conditions mentioned earlier [55]. The DLC% in the SLNs based on OA-loaded green IONPs was calculated using the following formula:

(2) $$DLC\%=\frac{OA_{entrapped}}{m_{total\;lipids}}\times 100\%$$

where $OA_{entrapped}$ is the weight of the entrapped OA in the lipid matrix, and $m_{total\;lipids}$ is the weight of the total lipids.

#### 2.2.2. Fourier Transform Infrared (FTIR) Spectroscopy Investigation of SLNs

In order to investigate the functional groups of the phytocompounds coming from both extracts (*Camellia sinensis* and *Ocimum basilicum*), and which are present on the IONPs’ surface, an FTIR spectroscopy investigation was performed. For this analysis, the Shimadzu Prestige-21 spectrometer (Duisburg, Germany) was used. The working conditions were as follows: room temperature at 22 ± 2 °C, a range of wavenumbers between 400–4000 cm^−1^, KBr pellets, and a resolution of 4 cm^−1^.

#### 2.2.3. Electron Microscopy Investigation

Using a Hitachi HD2700 with cold field emission gun STEM (Chiyoda, Tokyo, Japan) microscope, equipped with two windowless EDX detectors (X-Max^N^ 100) from Oxford Instruments (Oxford, UK), transmission electron microscopy (TEM) was carried out to analyze the size and shape of SLN test samples. For better conductivity and high-resolution TEM images, the SLN test samples were sputter-coated with carbon, which was previously mounted on a copper grid support and allowed to dry at room temperature (22 ± 2 °C). The micrographs were obtained by setting the microscope for the SLN analysis at an acceleration voltage of 200 kV. The elemental composition of the SLN test samples was assessed by EDX analysis, to identify the elements present in each sample and express them in weight percent (Wt%).

#### 2.2.4. Hydrodynamic Diameter and Zeta Potential

Dynamic light scattering (DLS) was performed to obtain the hydrodynamic diameter (Hd), the polydispersity index (PDI), and the zeta potential, using a Zetasizer Nano ZS (Malvern Instruments, Worcestershire, UK). Through photon correlation spectroscopy ranging from 0.4 nm to 9 μm, the particle size was measured, at 25 °C and 37 °C. Using the electrophoretic light scattering method, the zeta potential was measured, using a flow cell and distilled water as a dispersant, having a refractive index of 1.3329 and viscosity of 0.8879 cP.

### 2.3. In Vitro Culture Conditions

For the present study, a lung carcinoma cell line with epithelial-like morphology—A549 cells (CCL-185 from ATCC, LGC Standards GmbH, Wesel, Germany)—was used.

The complete cell culture media used for A549 cells was Dulbecco’s modified Eagle’s medium (DMEM, code 30-2002) supplemented with 10% fetal calf serum (FCS, code ATCC 30-2020) and 1% penicillin/streptomycin mixture.

The EpiAirway^TM^ in vitro models (Air-100, Lot 33251, Kit C) were purchased from MatTek Life Science Company, Ashland, MA, USA, and the microtissues were handled according to the manufacturer’s protocol (EpiAirway^TM^ AIR-100 Use Protocol).

The sterile conditions were respected for all in vitro protocols by using (i) a biosafety cabinet—MSC Advantage 12 (Thermo Fisher Scientific, Inc., Waltham, MA, USA)—and (ii) an incubator with a humidified atmosphere and 5% CO_2_ (Steri-Cycle i160 model from Thermo Fisher Scientific, Inc., Waltham, MA, USA).

### 2.4. Biosafety Evaluation Using EpiAirway^TM^ 3D In Vitro Inserts

To assess the biosafety profile of SLN test samples (SLN_CS 25@OA, SLN_CS 80@OA, SLN_OB 25@OA, SLN_OB 80@OA) at a concentration of 500 µg/mL, a human respiratory functional microtissue model—EpiAirway^TM^—was used. All the handling conditions were realized according to the manufacturer’s standard operation procedure (SOP), as described in detail in the study reported by Moaca et al. [53].

In brief, the inserts were quickly removed from agarose, wiped, and equilibrated overnight by incubating the inserts with assay medium (AIR-100-ASY) under standard conditions (37 °C, 5% CO_2_). Afterwards, the apical part of the inserts was rinsed twice with 400 µL TEER buffer, and the tissues were exposed to 50 µL of SLN test samples for 24 h. When the exposure period passed, each insert was washed, and after that, the MTT protocol was performed.

To quantify the viable rate of the tissues, the absorbance of each well was spectrophotometrically measured at two different wavelengths (570 nm and 650 nm) and a previously published formula was used [53].

### 2.5. Histopathological Assessment of 3D Respiratory Tissue Models (EpiAirway^TM^)

The histopathological analysis of the 3D EpiAirway^TM^ microtissues was realized by fixing the in vitro models in 10% formalin, embedding them in paraffin, and, after that, sectioning the tissues. A 4 µ thick formalin-fixed paraffin-embedded tissue sample was stained with hematoxylin and eosin (HE). The obtained slides were studied and photographed using the Leica DM750 microscope with a digital camera (with a magnification of 40× and objectives (×40 Ob.)).

### 2.6. Cell Viability Evaluation

The effect induced by SLN samples (SLN_CS 25@OA, SLN_CS 80@OA, SLN_OB 25@OA, SLN_OB 80@OA) at a concentration of 500 µg/mL on the A549 lung cancer cell line 24 h post treatment was analyzed using the Alamar blue colorimetric test, performed as previously described [56]. In brief, the cells were seeded onto 96-well plates to an initial density of 1 × 10^4^ cells/well and were cultured until the confluence reached 80%. Afterward, the cells were exposed to SLN samples; also, control cells were included, which were treated only with a culture medium. Quantification of the cell viability rate was determined by reading the absorbance at two different wavelengths (570 nm and 600 nm) using the xMark^TM^ microplate reader (Bio-Rad Laboratories, Hercules, CA, USA) and applying a previously published formula [57].

The Alamar blue method was selected to evaluate the cell viability rate of the A549 cell line after treatment with SLN samples because compared to another well-established method (MTT assay), the Alamar blue test shows superior sensitivity and colorimetric linearity depending on cell density, providing more accurate results [58].

### 2.7. Statistical Analysis

Results are expressed as mean ± standard deviation (SD) by comparing differences between sample-treated groups versus control employing the one-way ANOVA test followed by Dunnett’s post-test comparisons. The software used for data analysis was GraphPad Prism version 9.3.1 (San Diego, CA, USA). The statistically significant differences between data were marked with * (* *p* < 0.1; ** *p* < 0.01; *** *p* < 0.001; **** *p* < 0.0001).

## 3. Results

### 3.1. Physico-Chemical Characterization of SLN

#### 3.1.1. Entrapment Efficiency (EE%) and Drug Loading Capacity (DLC%) of the OA in the SLNs Based on OA-Loaded Green IONPs

The amount of the drug (OA) that was entrapped in the green IONPs and the drug content in the lipid matrix represent two important factors in the optimization of SLN-based OA-loaded green IONPs. The total amount of free OA determined by LC-MS in the case of green IONPs obtained from *Camellia sinensis* L. alcoholic extract samples was 195.5 μg for the SLN_CS 25@OA sample and 167.7 μg for the SLN_CS 80@OA sample. In the case of green IONPs obtained from *Ocimum basilicum* L. alcoholic extract samples, the total amount of free OA was 95.7 μg for the SLN_OB 25@OA sample and 63.8 μg for the SLN_OB 80@OA sample. After applying Equations (1) and (2) described above, the entrapment efficiency and drug loading capacity of OA within the lipid matrix were determined. The results are depicted in Table 1.

One can observe that the higher values of EE% were observed for samples prepared with green IONPs obtained starting from Ocimum basilicum L. alcoholic extract. Through the performed HPLC method, the entrapped efficiency of OA into the SLN matrix was found to range from 80.45 to 93.62% depending on the alcoholic extract used to obtain green IONPs. The drug loading of the SLNs based on OA-loaded green IONPs ranged from 1.226 to 1.427%, depending on the plant extract used (*Camellia sinensis* L alcoholic extract or *Ocimum basilicum* L. alcoholic extract) for the green IONP preparation.

#### 3.1.2. FTIR Characterization of SLNs

**Figure 2:**
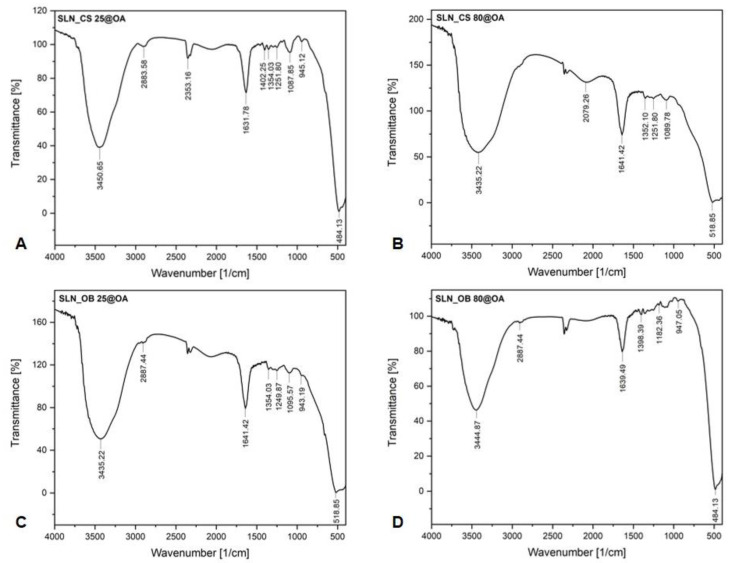
FTIR-spectra of SLNs: (**A**,**B**)—FTIR spectra of the samples containing OA-loaded green IONPs based on *Camellia sinensis* L. extract, at 25 °C and 80 °C; (**C**,**D**)—FTIR spectra of the samples containing OA-loaded green IONPs based on *Ocimum basilicum* L. extract, at 25 °C and 80 °C.

Figure 2 depicts the FTIR spectra of the SLNs based on OA-loaded green IONPs obtained starting from two alcoholic extracts of green tea (CS) and basil (OB), at 25 °C and 80 °C, respectively.

As can be noticed, there are no significant differences between the absorption bands of each sample. All FTIR spectra show the most important absorption maxima recorded. It is observed that the samples based on green IONPs obtained from the two extracts at 25 °C (Figure 2A,C) show more absorption maxima than the samples based on green IONPs obtained at 80 °C (Figure 2B,D). The first important and strong absorption bands are at 3450.65/3452.22 cm^−1^ recorded in the case of SLN samples based OA-loaded green IONPs obtained from *Camellia sinensis* L. extract, and at 3435.22/3444.87 cm^−1^ recorded in the case of SLN samples based on OA-loaded green IONPs obtained from *Ocimum basilicum* L. extract, which can be assigned to the O–H stretching vibration (hydroxyl groups (H-bonded)) present in water, alcohols, or phenols contained in the extracts of green tea and basil plant materials, as well as to N–H stretching in amines. The absorption band from 2883.58 cm^−1^ recorded in the case of the SLN_CS 25@OA sample, and the adsorption bands from 2887.44 cm^−1^ recorded in the case of the SLN_OB 25@OA and SLN_OB 80@OA samples, can be attributed to the saturated aliphatic C–H stretching bonds in alkanes, suggesting the occurrence of an aromatic ring attachment. The absorption peak from 2353.16 cm^−1^, recorded only in the case of the SLN_CS 25@OA sample, corresponds to the O=C=O stretching functional group. The absorption peaks located around 1600 cm^−1^, recorded in the case of all the SLNs samples, could be associated with the symmetric and asymmetric bending modes of C=O bonds of amino acid and esters, respectively, from the plant extracts, as well as to C=C stretching functional groups from the aromatic ring. The absorption peak from the 1402.25 cm^−1^ wavenumber, recorded only in the case of the SLN_CS 25@OA sample, revealed the presence of C–H bending from aromatic compounds and alkanes (methylene group). The absorption peaks recorded around 1300 cm^−1^, in all four spectra, are attributed to the C–H bending vibration of alkanes, referring to the binding of the aromatic ring –C–H for the in-plane bending absorption. The peaks recorded around 1200 cm^−1^, in the SLN_CS 25@OA, SLN_CS 80@OA, and SLN_OB 25@OA samples, corresponds to the aromatic acid ester C–O stretching vibration, most probably from the aromatic carbonyl acids present in both extracts. The absorption peak from 1182.36 cm^−1^, recorded only in the case of the SLN_OB 80@OA sample, can be attributed to the C–O stretching functional groups from esters or tertiary alcohols present in the extract. The peaks from 1087.85 cm^−1^, 1089.78 cm^−1^, and 1095.57 cm^−1^, recorded in the case of the SLN_CS 25@OA, SLN_CS 80@OA, and SLN_OB 25@OA samples, are assigned to the C–O stretching functional groups from aliphatic ethers or to the C–O stretching functional groups from primary and secondary alcohols. The absorption peaks around 900 cm^−1^, recorded in the case of SLN_CS 25@OA, SLN_OB 25@OA, and SLN_OB 80@OA, may correspond to the =C–H bending functional groups from alkenes or to the C=C bending monosubstituted functional groups from alkanes. The last absorption peaks recorded in all four FT-IR spectra, located in the inorganic domain (at 484.13 cm^−1^ in the case of the SLN_CS 25@OA and SLN_OB 80@OA samples, and at 518.85 cm^−1^ in the case of the SLN_CS 80@OA and SLN_OB 25@OA samples), correspond to the Fe-O stretching vibration from green IONPs.

#### 3.1.3. Electron Microscopy Investigations

**Figure 3:**
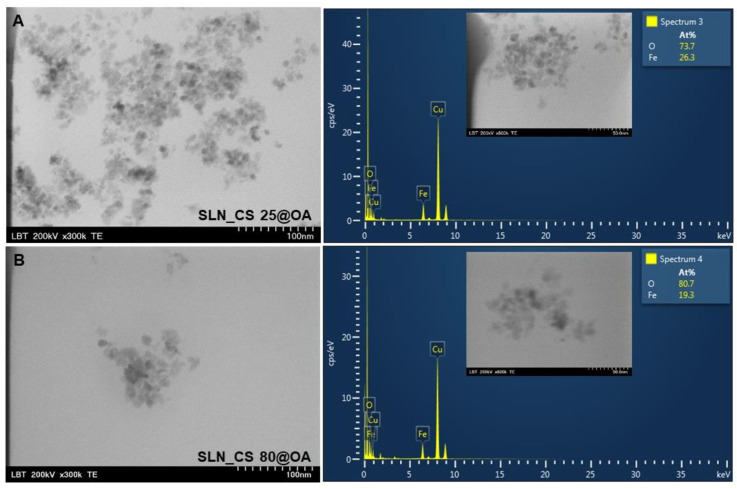
TEM images and EDX spectra of SLN based on OA-loaded with green-IONPs obtained from *Camellia sinensis* alcoholic extract at 25 °C (**A**) and at 80 °C (**B**).

**Figure 4:**
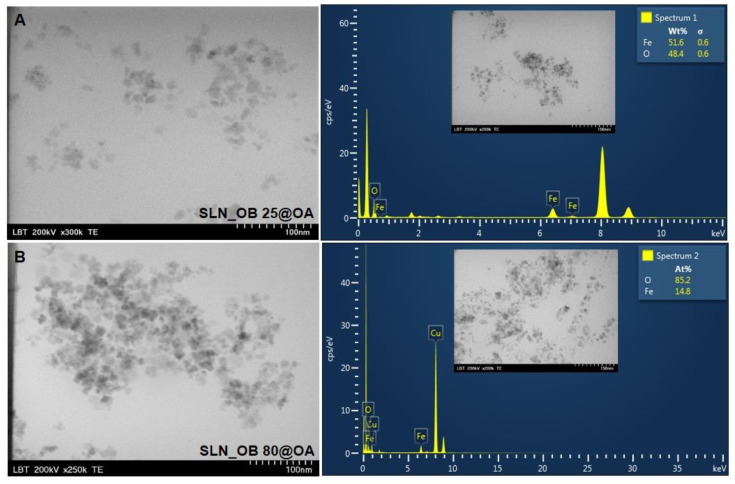
TEM images and EDX spectra of SLNs based on OA-loaded green-IONPs obtained from *Ocimum basilicum* alcoholic extract at 25 °C (**A**) and at 80 °C (**B**).

Figure 3 and Figure 4 depict the TEM images of the SLN test samples containing OA-loaded green-IONPs based on *Camellia sinensis* and on *Ocimum basilicum* plant materials, alongside their EDX analysis. The TEM images indicate that the SLN test samples composed of OA-loaded green-IONPs contain slightly agglomerated aggregates but are uniformly distributed. For the specific order of magnitude (300 kx), it can be seen that the SLN test samples were monodispersed with nearly spherical shapes and an average size between 7.26 and 15.84 nm. According to the TEM micrographs concerning the morphology and ultrastructure of SLN test samples fabricated from green-IONPs based on *Camellia sinensis* extract, it is observed that when green-IONPs obtained at 80 °C were used (Figure 3B), the aggregation of the SLN sample (SLN_CS 80@OA) decreased, and we observed more singular, nearly spherical solid lipid nanoparticles. On the contrary, in the case of SLN samples fabricated from green-IONPs based on *Ocimum basilicum* extract, the aggregation of the SLN sample occurred when it used green-IONPs obtained at 80 °C (Figure 4B), the SLN_OB 25@OA sample being formed by more singular, nearly spherical nanoparticles. Regarding the elemental composition of SLN test samples determined by EDX analysis, one can observe Fe and O as major components, alongside carbon and copper. These elements were identified by the peak amplitude and are expressed as atomic percentage values.

The carbon peaks identified in all EDX spectra are due to the tape used as grid support for SLN test sample immobilization, and copper might be due to the copper grid support on which the samples were fixed.

#### 3.1.4. DLS Measurements

The results regarding the values of the hydrodynamic diameter (Hd), polydispersity index (PDI), and zeta potential (ζ-potential) for all the SLN test samples recorded at 25 °C are described in Table 2.

The results recorded revealed the fact that the SLN samples based on OA-loaded green-IONPs have a narrow size distribution, being monomodal in nature (with a single population of nanoparticles), with a mean hydrodynamic diameter of 76.8 nm in the case of SLN samples based on green-IONPs obtained from *Camellia sinensis* extract and 70.7 nm in the case of SLN samples based on green-IONPs obtained from *Ocimum basilicum* extract. In addition, the measurement of the ζ-potential of each SLN sample indicated high stability of the solid lipid nano-structures against aggregation, with the values ranging from −20.7 to −38.5 mV. According to ζ-potential, it seems that the samples prepared with OA-loaded green-IONPs from *Ocimum basilicum* extract have a slightly higher stability than those prepared with OA-loaded green-IONPs from *Camellia sinensis* extract. This affirmation can also be observed from the polydispersity index (PDI).

### 3.2. Biosafety Profile Using EpiAirway^TM^ Microtissue

**Figure 5:**
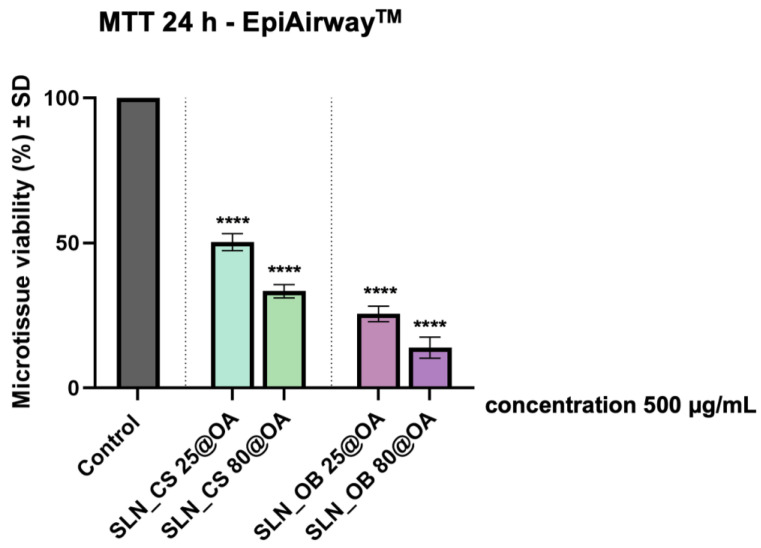
In vitro assessment of the biosafety profile of SLN samples at the level of a 3D normal in vitro bronchial model (EpiAirway^TM^, MatTek Company, Ashland, MA, USA) using an exposure interval of 24 h and a sample concentration that corresponds to 500 μg/mL green-IONPs. Statistically significant differences between the test and control groups were determined through one-way ANOVA analysis and Dunnett’s multiple comparison post-test (**** *p* < 0.0001).

To obtain an overview of the biosafety level of the four SLN samples, a healthy 3D human bronchial reconstructed tissue was employed for sample testing using an exposure interval of 24 h. At the level of EpiAirway^TM^ models, all the SLN samples induced a significant decrease in tissue viability. Thus, the lowest viability was recorded for SLN samples prepared from OA-loaded green-IONPs obtained from *Ocimum basilicum* extract (SLN_OB 25@OA and SLN_OB 80@OA). In this case, the 3D microtissues presented a viability of 25.54% when exposed to SLN_OB 25@OA, while SLN_OB 80@OA induced a viability rate of 13.93%. On the other hand, SLN samples prepared from OA-loaded green-IONPs obtained from *Camellia sinensis* extract induced lower toxicity—the microtissues treated with SLN_CS 25@OA manifested a viability of 50.30% and 33.35% when exposed to SLN_CS 80@OA, as presented in Figure 5.

### 3.3. Histopathological Evaluation of 3D Microtissues

**Figure 6:**
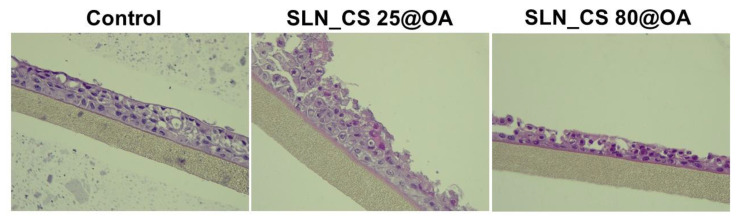
Morphological changes induced by SLN samples based on OA-loaded green IONPs prepared from *Camellia sinensis* L. extract at 25 °C and 80 °C, respectively, on bronchial respiratory tissue.

Figure 6 depicts the morphological aspects of the test sample-treated EpiAirway^TM^ 3D microtissues after exposure to the highest test concentration (500 µg/mL) of SLNs (SLN_CS 25@OA, SLN_CS 80@OA), composed from OA-loaded green-IONPs prepared from green tea (*Camellia sinensis*) extract.

One can observe that the EpiAirway^TM^ 3D models treated with SLN samples based on OA-loaded green IONPs obtained from *Camellia sinensis* L. extract at 25 °C (SLN_CS 25@OA) exhibit loose epithelial junctions. The superficial layer is denudated/loose from the surface epithelium integrity, the tissue appears with loose epithelial junctions, the epithelium appears without cilia, and the cells show apoptosis. Regarding the sample based on OA-loaded green IONPs obtained from *Camellia sinensis* L. extract at 80 °C (SLN_CS 80@OA), one can observe areas with the superficial denudation of the epithelium, epithelium without cilia, and goblet cells with loose junctions.

**Figure 7:**
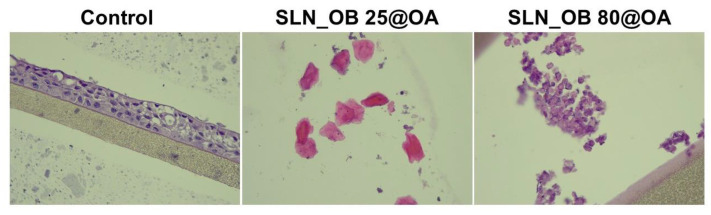
Morphological changes induced by SLN samples based on OA-loaded green IONPs prepared from *Ocimum basilicum* L. extract at 25 °C and 80 °C, respectively, on bronchial respiratory tissue.

Figure 7 depicts the morphological aspects of the test sample-treated EpiAirway^TM^ 3D microtissues after exposure to the highest test concentration (500 µg/mL) of SLNs (SLN_OB 25@OA, SLN_OB 80@OA), composed from OA-loading green-IONPs obtained from basil (*Ocimum basilicum*) extract.

Concerning the EpiAirway^TM^ 3D model treated with sample SLN_OB 25@OA, at 500 µg/mL, this shows apoptotic squamous cells and hyperkeratosis. The SLN_OB 80@OA sample at a concentration of 500 µg/mL induces autolysis on respiratory tract epithelium.

### 3.4. Cytotoxic Potential on A549 2D Cell Culture

**Figure 8:**
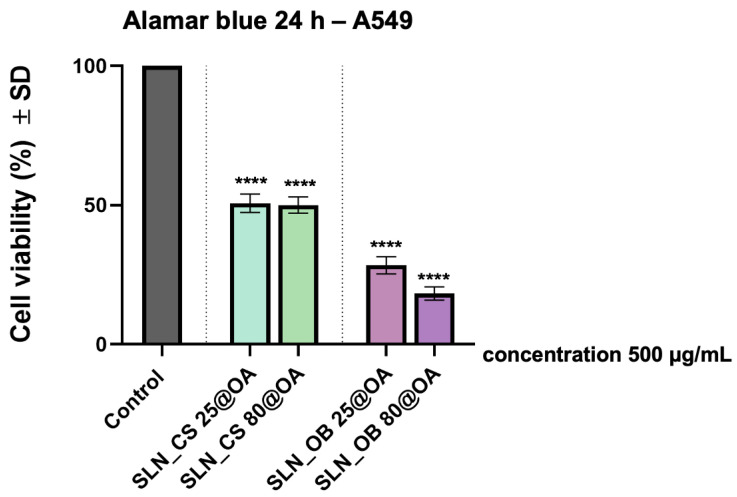
Viability percentages of human lung carcinoma A549 cells after treatment with SLN samples (SLN_CS 25@OA, SLN_CS 80@OA and SLN_OB 25@OA, SLN_OB 80@OA) at a concentration that corresponds to 500 μg/mL green-IONPs. One-way ANOVA analysis was applied to determine the statistical differences followed by Dunnett’s comparisons test (**** *p* < 0.0001).

To evaluate the toxicological potential of SLN samples, an in vitro model based on human lung carcinoma—A549 cells—was used, and the cytotoxic effect was assessed 24 h post exposure, using the Alamar blue colorimetric test. The results are presented in Figure 8, where it can be easily observed that the SLN samples based on OA-loaded green-IONPs obtained from *Ocimum basilicum* extract at 25 °C and 80 °C, respectively (SLN_OB 25@OA and SLN_OB 80@OA) induced a higher cell viability decrease, obtaining a rate of 28.38% for SLN_OB 25@OA sample and a viability rate of only 18.24% when the cells were treated with the SLN_OB 80@OA sample, which represents the lowest viability rate. On the other hand, both SLN samples based on OA-loaded green-IONPs obtained from *Camellia sinensis* extract induced similar viability rates, around 50%.

## 4. Discussion

Nanotechnology is considered a global hot topic in the research field, whether we refer to the chemical, agricultural, biological, or pharmaco-medical field [59]. The medical field is expected to be the most important beneficiary of the progress of nanotechnology; therefore, the use of structured nanomaterials offers a localized and targeted efficient alternative for diagnostics and treatment modalities of various diseases [60].

The current study highlights the fact that the most cited topics in the field of biomedical applications refer to plant-based synthesis. The plant materials employed to synthetize the IONPs were the leaves from two different plant extracts: *Camellia sinensis* L. and *Ocimum basilicum* L., obtaining four different green-IONPs at two different temperatures each (25 °C and 80 °C), with suitable features for biomedical applications, as follows: (i) favorable magnetic characteristics (high saturation magnetization and low/null coercive field); (ii) good biosafety profile on 3D EpiAirway^TM^ models (the microtissues expressing viability above 80% after treatment with 500 µg/mL green-IONPs, for 24 h); and (iii) high anti-tumoral activity, especially on A549 cells, as described in detail in the article recently published by our group [53]. The promising results obtained by our group in the aforementioned study constitute the starting point for the development of a nano-enabled pharmaceutical formulation based on green IONPs. In this regard, solid lipid NPs (SLNs) were selected as the most suitable type of nano-formulation due to their pharmacokinetics and bioavailability features [61], constituting also a delivery platform for hydrophobic drugs [9,62,63,64,65,66].

FT-IR spectroscopy investigation is a qualitative technique for the identification of the functional groups of the molecules contained by the pharmacological compounds in a plant extract [67]. The most relevant absorption peaks recorded in all the samples denote the presence of hydroxyl groups from water, alcohols, and phenols [68] present in both extracts, as well as the presence of amine functional groups contained by the green tea alkaloids (caffeine, theophylline, and theobromine) [69]. It was stated that the presence of hydroxyl groups (O–H) in plant extracts are assumed to be the phytocompounds responsible for the reduction of iron ions from FeCl_3_ [70]. In addition, the occurrence of the aromatic ring and alkyl group attachment was also highlighted through the peaks recorded around 2880 cm^−1^, which denote the presence of alkanes as well as the presence of the aromatic amines [71]. Aromatic compounds and alkanes (methylene group) are also highlighted by the peaks recorded between 1300 and 1400 cm^−1^. The absorption peaks recorded around 1600 cm^−1^ denote the presence of flavonoids, polyphenols, and catechins contained by the green tea extract [72], as well as the presence of amino acids (e.g., alanine, theanine, aspartic acid, arginine) [73]. A wavenumber around 1300 cm^−1^ also denotes the presence of the C–N stretch of amide-I in proteins contained by green tea extract [74]. The proteins from green tea extract have been found to be responsible for the reduction of iron ions [75,76]. At a wavenumber around 1200 cm^−1^ can be found the aromatic acid esters and/or aliphatic amines present in green tea extract [74], as well as the −C–O stretching vibration of the lignin structure present in basil extract [77]. The absorption peaks recorded between 1000 and 1180 cm^−1^ denote the presence of alcohols, ethers, carboxylic acids, and esters contained by both plant extracts [74,78,79]. In addition, the presence of green IONPs is also highlighted by the absorption peaks recorded in the inorganic domain at 484.13 cm^−1^ and 518.85 cm^−1^. These bands are assigned to the Fe-O stretching vibration from green IONPs. Our outcomes are also supported by the results obtained from other researchers [80,81,82].

For biological applications of the IONP-loaded solid lipid nanoparticles, some features related to their size and size distribution are important, as well as their shape, surface chemistry, stability, drug loading, and drug release at the targeted site. IONPs with a size range between 10 and 100 nm are accepted for biomedical applications, due to their longer circulation time in the organism and the ability to penetrate through very small capillaries as well as evade the reticuloendothelial system (RES). The advantages of using IONPs with smaller sizes are represented by increases in their surface efficiency to easily attach ligands or other molecules, having small settling velocities that give high stability in suspension and improve tissue diffusion [83]. Moreover, nanoparticles should be small enough to bypass the RES, because they are supposed to remain in circulation after injection and be able to pass through the capital systems, organs, and tissues, avoiding an embolus. In cell uptake, the size effectiveness of a nanomaterial depends also on the types of cells, because it is well known that each cell type has a different phenotype. Nanoparticle size is also important for obtaining an effect of improved permeability and retention. Nanoparticles larger than 10 nm may not penetrate the endothelium in physiological conditions but can enter pathological conditions, such as inflammation or tumors [84]. Nanoparticles smaller than 2 nm are not used for biomedical purposes. They can exhibit toxic effects because they diffuse through the cell membrane very quickly, causing intracellular damage. On the other hand, if IONPs have a larger size (above 200 nm), those can be processed by phagocytic cells of the organism or can be concentrated in the spleen after they are removed by mechanical filtration from the bloodstream. IONPs with smaller sizes (under 10 nm) are rapidly removed by renal clearance and extravasation. Regarding the shape of IONPs, it was stated that rod-shaped, worm-shaped, or bead-shaped IONPs have a longer blood circulation time in living organisms as compared to the spherical ones. It is believed that this fact can be due to the lower phagocytic activity of macrophages caused by rod-shaped NPs [85]. Spherical IONPs are the most desired for biomedical applications because their surface can be conjugated with various ligands, proteins, surfactants, polymers, and even drugs, for a better release at the targeted site as well, leading to an increased cellular toxicity. To prolong the time spent in the circulatory system, IONPs must possess the antifouling property, meaning that the attachments used as coating agents are not to be adsorbed on the surface of IONPs [86]. The coating process increases the colloidal stability and improves the dispersity of IONPs in aqueous medium. Uncoated IONPs (naked) can easily oxidize in ambient conditions, causing the agglomeration process, and thus rapid total clearance by the RES takes place [87].

The results obtained herein for the four types of SLNs indicated good physicochemical characteristics as regards size, shape, and morphology, as well as hydrodynamic diameter and zeta potential (stability). Figure 3 and Figure 4 depict the TEM images of as-prepared SLN samples, which show a slight agglomeration of the nanoparticles in the case of the sample SLN_CS 25@OA and the case of sample SLN_OB 80@OA. The other two SLN samples (SLN_CS 80@OA and SLN_OB 25@OA) are observed as more singular, nearly spherical solid lipid nanoparticles with a smooth surface. This investigation is well correlated with the TEM analysis of green-IONPs used for the preparation of SLN samples reported and discussed in reference [53]. We believe that in the case of SLN samples based on OA-loaded green IONPs obtained with *Camellia sinensis* extract, nanoparticle agglomeration increased with the reaction temperature because green tea contains a variety of polyphenolic compounds which degrade when the temperature is increased. EDX analysis reveals the presence of Fe and O as the major elements in all the SLN samples. Besides these two, copper was also identified. This element may come from the grid support, considering that the oxygen was reported in a higher amount than iron, possibly due to the copper of the oxidized grid. The size, zeta potential, and polydispersity index of SLN test samples are summarized in Table 2. According to the results, in the case of SLN samples based on OA-loaded green IONPs obtained from *Camellia sinensis* extract, the z-average is almost the same, regardless of the temperature employed for the green IONPs obtained. A slightly significant difference could be observed in the case of SLN samples based on OA-loaded green IONPs obtained from *Ocimum basilicum* extract, which can be attributed to the fact that, in this case, the nanoparticles are agglomerated, and the oleanolic acid (OA) loads several nanoparticles that are attracted to each other by strong van der Waals forces, instead of a single nanoparticle. However, the results indicated a monomodal particle size distribution, which suggests the presence of one population of green IONPs in each SLN test sample. The zeta potential of the SLN samples prepared with green IONPs from *Ocimum basilicum* extract was slightly better than those prepared with green IONPs from *Camellia sinensis*, but even so, the values obtained show that all the samples possess stability over time. The colloidal stability of IONPs is linked to the loading surface of the nanoparticles and their biodistribution. Surface charging is described by the nature and behavior of surface groups in the solution (at a given pH) and in the presence of an electrolyte. A high value of zeta potential is an indication of stability, due to electrostatic interaction. This affirmation comes in agreement with data in the literature [88], in which it is specified that ζ-potential values below −30 mV or the values above +30 mV ensure good stability to the entire system, due to the strong electrostatic repulsion between nanoparticles. But, at the same time, a ζ-potential value near 0 mV leads to the flocculation of the entire system and thus IONP sediments. In addition, there are reported studies that have demonstrated that a positively charged surface of IONPs leads to increased toxicity in the biomedical field [89,90,91].

However, the biological evaluation of the biosafety profile of SLN test samples revealed some concerns when applied at a concentration corresponding to 500 µg/mL green-IONPs on EpiAirway^TM^ microtissues. Even though, as already mentioned above, the green-IONPs obtained from both plant materials (green tea and basil) presented good biosafety profiles on 3D bronchial models [53], it seems that the SLN samples applied at the same corresponding concentration of green-IONPs (500 µg/mL) induce a significant viability decrease of EpiAirway^TM^ microtissues, especially the SLN samples based on OA-loaded green-IONPs obtained from *Ocimum basilicum* extract, which showed the viability of 25.54% when treated with SLN_OB 25@OA sample and viability of 13.93% after exposure to the SLN_OB 80@OA sample, as presented in Figure 5. This aspect may be correlated with an unbalanced ratio between the amount of blank SLNs and green IONPs used in the formulation process, which led to a very high concentration of SLNs in the final preparation. Thus, when the final sample of SLNs is applied at a concentration corresponding to 500 µg/mL of green-IONPs, a very large amount of SLNs is applied, too, which could cause harmful effects on 3D microtissues and 2D culture cells. This statement is also reinforced by the drug loading capacity measured at 25 °C, which proved to be 1.226% and 1.269% in the case of SLN samples obtained starting from green IONPs obtained from *Camellia sinensis* L. extract, at 25 °C and 80 °C, and 1.379% and 1.427% in the case of SLNs samples obtained starting from green IONPs obtained from *Ocimum basilicum* L. extract, at 25 °C and 80 °C. In addition, another cause could be the entrapment efficiency of oleanolic acid on the surface of green IONPs, which proved to be quite high in the case of SLNs based on green IONPs obtained from basil extracts (90.43% for SLN_OB 25@OA sample and 93.62% for the SLN_OB 80@OA sample) as compared with the SLNs based on green IONPs obtained from green tea extracts (80.45% for SLN_CS 25@OA sample and 83.23% for the SLN_CS 80@OA sample).

Nevertheless, to understand the effect induced by SLN samples on the 3D bronchial models, a histopathological assessment of the microtissues was performed. The outcomes showed that the morphological changes that occurred imply major changes in the multilayered respiratory tract epithelium, such as loss of the surface epithelium integrity, loss of epithelial junctions, loss of cilia, hyperkeratosis, and cell death caused by apoptosis.

For biological assessment, in vitro models are preferred instead of in vivo models due to several advantages presented by the cell lines, such as controllable parameters, facile data interpretation, reduced expenses, and the overcoming of ethical regulations related to animal use for research. Therefore, in the current study, the cytotoxic impact of SLN samples was evaluated on a human lung carcinoma A549 cell line. This in vitro model was selected due to the promising results obtained for the green-IONPs used to obtain the four SLN samples, when applied at concentrations up to 500 µg/mL on this particular cell line (A549), compared to another lung large cancer cell line (NCI-H460) [53]. Thus, the present results indicated that SLN samples based on OA-loaded green-IONPs obtained from *Ocimum basilicum* extract induced high anti-tumor activity on A549 cells, inducing a viability rate of 28.38% when the SLN_OB 25@OA sample was applied. In comparison, a viability rate of only 18.24% was recorded when A549 cells were exposed to the SLN_OB 25@OA sample using a concentration that corresponds to 500 µg/mL of green IONPs for the same interval time, as shown in Figure 8. Also, as an active therapeutic structure, oleanolic acid (OA) was selected for its anticarcinogenic potential [92,93], knowing to interfere with different pathways of lung cancer development, such as DNA fragmentation and caspase-3 activation of non-small lung cancer cell lines (A549 and H460) possessing multi-drug resistance proteins [51]; mitophagy via a Parkin independent pathway in A549 cells [94]; or cell cycle arrest of lung cancer cells through miR-122/Cyclin G1/ MEF2D pathway [95]. Therefore, due to its high anticarcinogenic activity, the SLN formulations were applied in the present study on the A549 cell line in a concentration that corresponds to 25 µg/mL OA (and 500 µg/mL green-IONPs), showing high cytotoxicity on human lung carcinoma cells. Most likely, the cytotoxic effects observed after the application of the test compounds, both on the A549 cell line and on the 3D bronchial tissues, are due to a synergistic effect created by the entire lipid matrix, formed by green IONPs and the amount of drug entrapped on their surface. Our group of researchers was shown that the iron oxide nanoparticles obtained by green synthesis, starting from an aqueous extract of *Artemisia absinthium* L. plant material, have a cytotoxic effect on human amelanotic melanoma cells (A375) and human epidermoid carcinoma cells (A431) after treatment with wormwood leaves and stems extracts. The A375 cells were significantly affected by the test compounds (cytotoxicity rate of 23.73% after exposure to 500 μg/mL of green-IONPs obtained from wormwood leaves extract, and a cytotoxicity rate of 20.06% after exposure the cells to the same dose of green IONPs obtained from wormwood stems extract). Even the A431 cells were affected by the green IONPs obtained from leaves and stems of wormwood, but in a slight manner as compared to the melanoma cell line (cytotoxicity rate ranging from 5 to 10%) [96]. In addition, it was shown that the tumorigenic cells released a high amount of extracellular LDH and specific signs of apoptosis when exposed to a concentration of 500 μg/mL of green IONPs for an interval of 72 h. Moreover, in another research study reported by our team, it was reported that the green IONPs obtained from *Camellia sinensis* L. extract at 80 °C induce a viability percentage of A549 cells between 71.5 and 70% at a concentration of 500 μg/mL, the viability decreasing in a time-dependent manner [53]. As regards the anticancer potential of oleanolic acid, Zhao and co-workers [95] found that oleanolic acid suppresses the proliferation of A549 lung carcinoma cells in time and in a dose-dependent manner (~40% proliferation rate at a concentration of 60 μg/mL OA). In addition, Song and co-workers reported the cytotoxic effect of oleanolic acid as compared with an OA derivate (SZC017) on the A549 human lung cancer cell line [97]. The results obtained by the authors showed that the OA derivate significantly decreased cell viability in A549 cells as compared with oleanolic acid, for which the inhibitory effect on cell viability in A549 cells was around 60% at a concentration of 40 μM OA. The same results were obtained also by Castrejón-Jiménez and co-workers [94], in a study in which they investigated the anticancer activity of ursolic and oleanolic acids on the viability of A549 human lung cancer cells and the role of autophagy in their activity. The authors found that a lower concentration of oleanolic acid (5 μg/mL) did not cause a cytotoxic effect, while 24–48 h post treatment, 40 μg/mL oleanolic acid caused a notable decline in A549 cell viability (56%) [94]. In another study, Wang and co-workers [98] investigate the anti-proliferative activity of triterpenoids and sterols isolated from *Alstonia scholaris* leaf extract against non-small-cell lung (NSCL) carcinoma cells. The authors managed to isolate eight triterpenoids and five sterols from the hexane portion of *A. scholaris* leaf extract, among which was oleanolic acid. Contrary to the above-mentioned studies, the authors reported that the oleanolic acid (15.1%) isolated from the *Alstonia scholaris* leaf extract did not possess any anti-proliferative activity against A549 cells [98]. Therefore, studies in the specialized literature report an average cytotoxic effect of oleanolic acid of 40% (at a tested concentration higher than the concentration used in the present study: 40 μg/mL vs. 25 μg/mL) on the A549 tumor cells. This means that the values regarding the cytotoxic effect obtained for the SLN samples tested in the present study (cell viability rate of 28.38% for the SLN_OB 25@OA sample and 18.24% for the SLN_OB 80@OA sample) were due to the synergistic effect of the entire lipidic matrix. This statement is supported by a recent study carried out by Abulaiti and co-workers [99], in which the authors embedded oleanolic acid in a chitosan nano-complex and tested the anticancer potential of the conjugated nano-complex (OAC) on A549 human lung cancer cells. The authors’ results showed that the oleanolic acid entrapped in the chitosan polymeric matrix changes the cells’ morphology, restrains cell development, and prompts apoptosis in A-549 cells, the cell apoptosis being predominantly accompanied by nuclear material fragmentation in the treated A549 cells. Thus, the authors portrayed the anticancer efficacy of oleanolic acid-conjugated chitosan nanoparticles as an improved synergistic drug system for cancer therapy [99].

According to ISO Standard 10993-5:2009 which represents the standard for the biological assessment of medical devices [100], when a test sample induces a viability rate under 70%, it is considered cytotoxic. Therefore, the SLN obtained in the current study (SLN_CS 25@OA, SLN_CS 80@OA, SLN_OB 25@OA, and SLN_OB 80@OA) may be labeled as cytotoxic when applied at concentrations up to 500 µg/mL of green-IONPs and 25 µg/mL OA for 24 h on A549 monolayer. In summary, the present study provides preliminary data on the biological effects of SLN-based OA-loaded green IONP behavior under normal conditions on A549 human lung carcinoma in terms of its cytotoxicity, as well as on 3D functional microtissues obtained from normal bronchial cells, cultured at the air–liquid interface (EpiAirway^TM^ model), regarding its biosafety profile. However, comparing the biosafety profile and the cytotoxicity activity of the SLN samples, it can be observed that only the SLN_CS 25@OA sample showed similar viability of healthy bronchial tissue to that expressed by human lung carcinoma A549 cells (a viability rate of around 50%), whereas for the other three SLN samples (SLN_CS 80@OA, SLN_OB 25@OA, SLN_OB 80@OA), the viability of the healthy EpiAirway^TM^ models is more decreased compared to the viability of the A549 cells, an aspect that may raise toxicological questions for human health, since healthy tissue is more affected than tumorigenic cells. This aspect may be correlated with the nanoparticle’s degree of agglomeration that may influence the oleanolic acid (OA) loads, which may also be attracted to each other via strong van der Waals forces. Therefore, at this stage, only SLN_CS 25@OA could be considered safe enough for future development addressing lung cancer treatment. Nevertheless, for the other three SLN samples, further investigations are needed regarding the optimization of the SLN’s manufacturing recipe by employing computational prediction of the ADME TOX profile using advanced software tools, such as SwissADME (http://www.swissadme.ch, accessed on 5 January 2024) or FAFDrugs (http://fafdrugs4.mti.univ-paris-diderot.fr, accessed on 5 January 2024) [101], as well as on adjusting their dose to increase the biosafety level, so that they could be further used as preliminary data for future in vivo experiments.

## 5. Conclusions

The current study reports a facile one-step method to obtain a new pharmaceutical formulation—solid lipid nanoparticles (SLNs)—by employing four different green synthesized IONPs, which all possess suitable physicochemical features for biomedical applications. Hence, electron microscopy investigations exhibited that the formed SLN samples have a nearly spherical shape with a narrow size distribution in the range of 7–15 nm, being monomodal in nature, a value that could indicate the presence of high polydispersity of green-IONPs in the structure of the samples. These results follow the data obtained from DLS measurements. In addition, the SLN samples have a negative zeta potential, which denotes a high stability of the solid lipid nano-structures against aggregation. The formed green IONPs were loaded with a pentacyclic triterpene—oleanolic acid, resulting an entrapped efficiency into green IONP surfaces, which ranged from 80.45 to 83.23% in the case of SLN samples containing green IONPs obtained from *Camellia sinensis* L. extract and an entrapped efficiency that ranged from 90.43 to 93.62% in the case of SLN samples containing green IONPs from *Ocimum basilicum* L. extract. The drug loading capacity was also higher in the case of SLNs based on OA loaded with green IONPs obtained from basil extracts (1.379% for the green IONPs obtained at 25 °C and 1.427% for the green IONPs obtained at 80 °C) as compared with SLNs based on OA loaded with green IONPs obtained from green tea extracts (1.226% for the sample obtained at 25 °C and 1.269% for the sample obtained at 80 °C). Further, the anticancer potential of the SLNs on lung cancer cells was evaluated. All four samples possess high anti-tumor activity, especially the SLN samples based on OA-loaded green-IONPs prepared from *Ocimum basilicum* extract, regardless of the temperature at which the nanoparticles were synthesized (25 °C and 80 °C). Nevertheless, the biosafety profile of all four samples should be further investigated, and based on the obtained results, the synthesis process should be adjusted accordingly.

Finally, the findings reported could be important contributions to the development of a new and innovative lung cancer therapeutic approach. Therefore, future directions regarding the advanced screening of these multifunctional nanostructures are considered, including targeted drug delivery, with the help of green IONP magnetic behavior.

## Figures and Tables

**Table 1:** EE% and DLC% of OA in the SLNs based on OA-loaded green IONPs.

Sample	Free OA [μg]	EE [%]	DLC [%]
SLN_CS 25@OA	195.5	80.45	1.226
SLN_CS 80@OA	167.7	83.23	1.269
SLN_OB 25@OA	95.7	90.43	1.379
SLN_OB 80@OA	63.8	93.62	1.427

**Table 2:** Characteristics of SLN test samples recorded at 25 °C.

Test Sample	Hd (nm)	PDI	ζ-Potential (mV)
SLN_CS 25@OA	75.3	0.283	−29.2
SLN_CS 80@OA	78.2	0.366	−38.5
SLN_OB 25@OA	66.5	0.189	−20.7
SLN_OB 80@OA	74.8	0.276	−28.4

## Data Availability

Authors can provide raw data under request.
